# Quasi-Static Mechanical Response of Density-Graded Polyurea Elastomeric Foams

**DOI:** 10.1021/acsapm.3c00062

**Published:** 2023-03-29

**Authors:** Mark Smeets, Behrad Koohbor, George Youssef

**Affiliations:** † Experimental Mechanics Laboratory, Mechanical Engineering Department, San Diego State University, 5500 Campanile Drive, San Diego, California 921821, United States; ‡ Department of Mechanical Engineering, Rowan University, 201 Mullica Hill Road, Glassboro, New Jersey 08028, United States

**Keywords:** density gradation, elastomeric foams, polyurea foams, multilayer gradation, loading efficacy

## Abstract

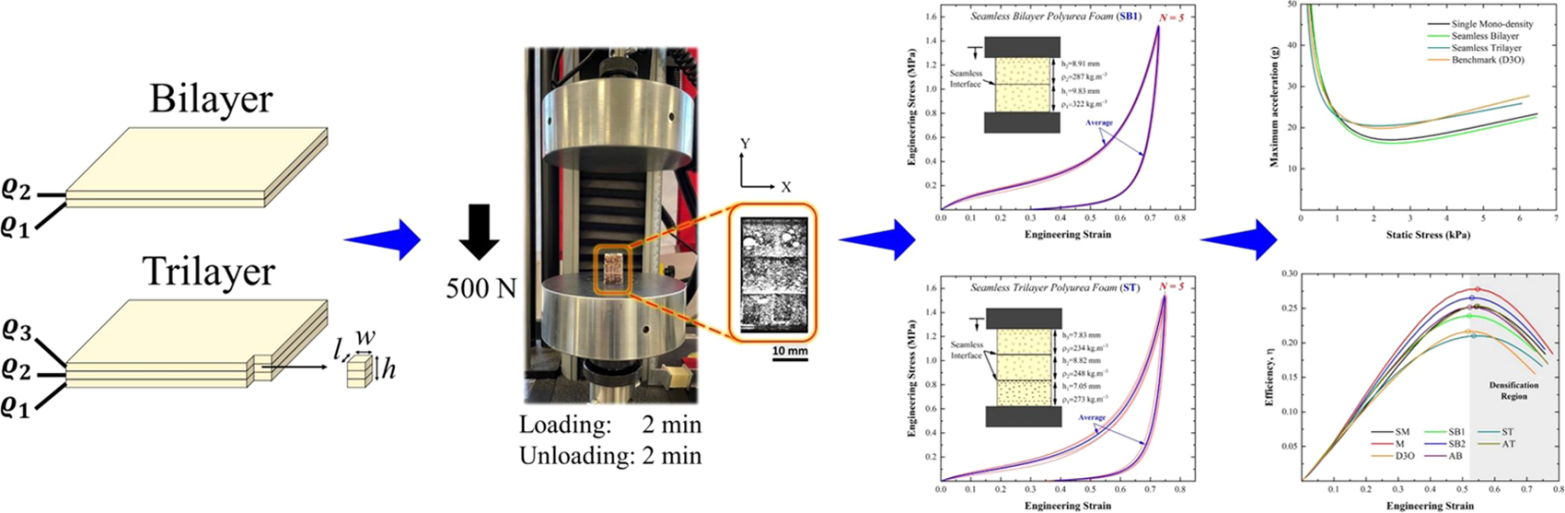

Density gradation of foam structures has been investigated and found to be a practical approach to improve the mechanical efficacy of protective padding in several applications based on nature-based evidence of effectiveness. This research aims to disclose a discrete gradation approach without adhesives by relying on the properties of the frothed foam slurry to bond and penetrate through previously cured foam sheets naturally. As confirmed by electron microscopy observations, bilayer- and trilayer-graded elastomeric polyurea foam sheets were fabricated, resulting in seamless interfaces. The mechanical performance of seamless, graded foam samples was compared with monolayer, mono-density benchmark foam, considered the industry standard for impact mitigation. All foam samples were submitted to compressive loading at a quasi-static rate, reporting key performance indicators (KPIs) such as specific energy absorption, efficiency, and ideality. Polyurea foams, irrespective of gradation and interface type, outperformed benchmark foam in several KPIs despite the drastic difference in the effective or average density. The average compressive stress–strain curves were fitted into empirical constitutive models to reveal critical insights into the elastic, plateau, and densification behaviors of the tested foam configuration. The novelty of these outcomes includes (1) a fabrication approach to adhesive-free density-graded foam structures, (2) implementation of a diverse set of KPIs to assess the mechanical efficacy of foams, and (3) elucidation of the superiority of polyurea foam-based lightweight protective paddings. Future research will focus on assessing the dynamic performance of these graded foam structures under impact loading conditions at a wide range of velocities.

## 1 Introduction

The eager pursuits of protecting humans, packages, and structures from severe mechanical loads highlight several imperative requirements, the prime of which is shunting the repeated force imposition, irrespective of the strain rate. A symbiotic criterion is reducing the weight of these protective gears to subdue the inertial effects resulting from increasing the mass of the protected objects. The low weight requirement is also critical from optimal space utilization and logistics perspectives. Axiomatically, the quest for an effective protective structure hinges on advancement to ordered and stochastic polymeric foams (i.e., cellular solids) given their desirable mechanical behavior at highly reduced weight penalty.^1^ Notably, the mechanical performance, defined herein broadly to encompass the elastic, plateau, and densification regions within the stress–strain response of cellular solids, strongly depends on their relative density (, where ϱ^*f*^ is the foam density and ϱ^*s*^ is the density of the bulk material).^2^ The importance of the relative density, and, in turn, its relation to the mechanical efficacy of the foam resulted in the emergence of the density gradation approach to achieve strategic and localized performance, such as absorbed-energy-to-weight ratio.^3^

The premise of density gradation is coded in natural materials, where biology has perfected this concept to achieve competitive properties that engineered materials seek to mimic. Density gradation is ubiquitous in naturally occurring cellular structures, including tubercular bone in humans,^4^ bamboo, pomelo, and palm plant stems,^5^ the exoskeleton of crustaceans,^6^ cork,^7^ and butterfly wings,^8^ to name a few common examples.^9^ The prime advantage of gradation is enhancing the property map of cellular solids, irrespective of the type of base material, by improving the mechanical, acoustic, and thermal properties while allowing vertical and lateral tailorability of the structure to mitigate incoming forces at a reduced weight penalty.^3^ It can then be thought that density gradation is the second generation of technological advancements in cellular structure. The ongoing third generation of density gradation is powered by parallel progress in additive manufacturing with unprecedented potential to locally tune the structure at levels unattainable previously,^3,10^ achieving exceptional mechanical performance.^11^ The latter is exemplified in enhanced energy absorption,^12^ auxeticity,^13,14^ and toughness.^15^ The reader is referred to this topical review^10^ for a comprehensive overview of the state-of-the-art of application of additive manufacturing to cellular solids, a contemporary but peripheral topic to this research. Conventional approaches to fabricate density-graded polymeric and metallic foams have also been reported, including the approaches developed by Gupta et al.,^16^ Cusson et al.,^17^ Yuan et al.,^18^ and Elsing et al.^19^ The utility of density-graded tailored structures has transpired in real-life applications,^3^ extending beyond academic and laboratory testing.

The quasi-static and dynamic behaviors of density-graded polymeric foams have been vigorously investigated and previously reported.^3,20^ In addition, several analytical and numerical modeling schemas have been proposed to optimize the gradation strategy in favor of enhanced energy absorption capabilities.^3,21^ Several recent hybrid experimental and analytical investigations focused on expanded polystyrene and rigid polyurethane foams, given their ubiquity in practical applications, including liners of protective sports helmets and cushioning protection of electronic packages.^21,22^ For example, Li et al. proposed a constitutive model to describe the compressive stress–strain behavior of expanded polystyrene foam based on the model by Schraad and Harlow.^21^ They relied on definitions of (i) strain-based transition zones of the triphasic behavior of cellular foams embodied in the elastic, plateau, and densification regions and (ii) piecewise geometrical stiffness parameters corresponding to the behavioral phases.^21^ Li et al. also accounted for the strain rate effect on the mechanical response of functionally graded polymeric foams, extending the applicability of their model to the moderate strain rate testing regime. Li et al. demonstrated that their analytical model could accurately predict the averaged compressive stress–strain relations of graded foam structured at strain rates up to ca. 10^2^ s^–1^.^21^ Focusing on the quasi-static and dynamic behavior of rigid polyurethane graded foams, Koohbor et al. presented a comprehensive experimental study while emphasizing the effect of positive and negative gradation on the overall mechanical behavior using split Hopkinson bar experimental testing setup and full-field strain measurements.^23^ Koohbor and collaborators elucidated different deformation mechanisms based on the loading rate and gradation configuration, demonstrating that positive gradation (i.e., the lower-density layer facing the incoming impact object) is more suitable for impact mitigation applications.^23^

In recent years, polyurea foams emerged in the scientific literature as a suitable material candidate for biomechanical impact mitigation applications.^3,24−36^ Such emphasis was motivated by the contemporary work of Reed et al.^26,29^ and Ramirez et al.,^34−36^ reporting viable approaches to foam bulk polyurea using spontaneous foaming and heat-activated/controlled foaming methods, respectively. Polyurea is a fascinating class of polymers with several formulations that span the spectrum from linear to cross-linked polymers.^37−41^ Polyurea, with a specific mixture of diamine and diisocyanate, found favor in the scientific and technological communities for its superior mechanical and thermal properties, including hygrothermal stability,^42^ large extensibility,^43,44^ a broad range of operating temperature,^32,45^ adhesive properties,^46−48^ chemical, moisture, and abrasion resistance, and toughness based on its tailored segmental microstructure.^49−51^ This polyurea formulation was also found to be mildly affected by extended exposure to ultraviolet radiation.^43^ Research on bulk polyurea for impact mitigation applications, with an emphasis on military infrastructures and assets, i.e., response to ultrahigh ballistic strain rate, was recently collated by Barsoum.^52,53^ The latter research culmination showed the momentous promise of this material while signifying polyurea as an interesting scientific testbed. Realizing the superior performance of polyurea while acknowledging that cellular solids inherit their aggregated properties from their bulk native material, Reed et al.^26,29^ and Ramirez et al.^34,35^ independently sought to fabricate polyurea foams. Thus far, these groups have vigorously investigated polyurea foams as a function of loading rate, temperature, and operating conditions,^3,24−36^ substantiating the stated supposition. The foaming method reported by Reed and collaborators is more conducive to creating adhesive-free multilayer density-graded sheets, as in the current report.

From a mechanics perspective, the hyper-viscoelastic properties of bulk polyurea are also embodied in the foamed version,^26,29^ resulting in sustaining single and repeated impacts at different energies.^33,54,55^ Polyurea foams investigated herein have been classified as self-reinforced, semiclosed cellular solids based on a twofold rationale. First, polyurea microspheres nucleate during the violent mixing step to froth the chemical constituents in the water mixing solution based on the precipitation polymerization approach.^27,30,32^ The emulsified polyurea microspheres are then deposited on the internal surfaces of the cells during the water-draining step, effectively reinforcing the cells without unnecessarily increasing the wall thickness, i.e., without substantially increasing the density.^56^ The close match of the properties of the polyurea microspheres and the surrounding polyurea foam cells, i.e., made of the same materials, negate interfacing issues common to particulate composites. Second, the microstructure of polyurea foams, based on that reported by Reed et al., exhibits a combination of small, closed cells surrounding relatively larger and perforated cells.^24,26,27^ Do et al. rationalized the process–structure interrelationship based on scanning electron microscopy (SEM) analysis, in addition to the mechanical behavior previously reported by Reed et al.^24,26,27^ Since these initial reports, the mechanical performance of polyurea foams has garnered additional attention, with a few case studies pertaining to biomedical applications.^28,29,34^ Of particular interest is our recent report on the behavior of positive gradation polyurea foams, assembled using relatively thick adhesive (ca. 1 mm) with a wide range of mechanical properties. Uddin et al. used full-field measurements in quasi-static and drop impact loading scenarios and showed that the adherent could be used to tailor the mesoscale mechanical behavior of bilayer polyurea foam structure, where the stiff adhesive has an adverse effect on the efficiency while a compliant counterpart has a favorable influence.^54^ While comprehensive, the study by Uddin et al. posed insightful inquiry about the type and thickness of the adhering layer, presenting two “what if” scenarios: (i) what if the multilayer positively graded foam samples are assembled natively without any adhesives, and (ii) what if the adhesive is replaced by microscale thickness bulk polyurea layer. These conjectures are the prime motivations of the research leading to this report.

At the essence of the motivating conjectures is the implication of the current challenge in manufacturing multilayer-graded foam structures, namely, the interface adhesion between subsequent layers. It is well known that polymeric foams can be made in a wide range of densities, even with very small discrete increments, allowing quasi-continuous gradation. However, this discrete gradation approach is faced with a practicality challenge (e.g., the labor associated with the fabrication of individual layers and adhering these layers in a specific configuration). The magnitude and extent of this challenge are eclipsed by the fundamental issue of adhesion even if different mechanisms are used, i.e., mechanical, physical, or chemical.^21,57,58^ In general, the interface properties play a deciding role in the overall performance of graded foams.^54^ Here, investigating polyurea foams presents an opportune pathway to resolve the adhesion issue in graded foams since the base material has been reported to be an excellent adhesive. For example, polyurea adhesive increased the interfacial strength of *E*-glass composite joints nearly twofold compared to adhesion with traditional epoxy.^48^ Furthermore, the starting point of the foaming process, as discussed next, entails pouring a frothed polyurea slurry in a mold coated with a release film, facilitating the production of multilayer self-adhered graded foam sheets through a strategic sequential pour of tailored slurries. The sequential pouring approach to create multilayer, elastomeric, graded polyurea foams is introduced here for the first time, where the behavior of these graded samples is compared to adhered counterparts to resolve the adhesion–performance interdependence.

The objective of the research leading to this paper is to elucidate the mechanical behavior of multilayer, graded elastomeric foams under quasi-static loading conditions. The stress–strain responses are compared to forecast the dynamic behavior of these structures using the foam efficiency metric. A semiempirical constitutive model, i.e., Avalle model,^59^ is used to synthesize the experimental results, summarizing the homogenized properties of density-graded polyurea elastomeric foams. A brief biomechanical case study is also presented to substantiate the utility of graded polyurea foam for biomechanics impact mitigation scenarios.

## 2 Materials and Methods

This section is divided into three subsections, including the sample fabrication process starting from sheets of mono-density foam to multilayered density-graded specimens. In the second subsection, the experimental characterization approach is discussed in detail. The final subsection is dedicated to the analysis approach and the semiempirical model used herein.

### 2.1 Materials and Samples Preparation

Two density-graded sample configurations were fabricated and characterized in this research, namely, adhesive- and naturally assembled configurations. All samples were made of elastomeric polyurea foams in-house. Irrespective of the final density gradation configuration, polyurea foam sheets were cast by mixing modified methylene diisocyanate (Isonate 143L MDI, Dow Chemical) with oligomeric diamine (Versalink P1000, Evonik) using a 1:4 weight ratio, respectively. These constituents were violently mixed in deionized water, resulting in a frothed foam slurry due to the emulsion of chemicals in water and the production of carbon dioxide from the reaction between the mixing solvent and diisocyanate. Before casting the foam in Teflon-coated aluminum mold, the excess water was drained without allowing the floating foam slurry to escape the mixing container. The size of the aluminum mold was 30 cm × 30 cm, while the thickness was adjusted using spacers between the mold walls and the polyethylene cover to tube the desired foam density and height.^26^ Each foam layer was cured at ambient conditions in the fully assembled mold for 24 h, followed by a 48 h dehydration period in the same conditions with the cover removed or the sheet completely demolded. The rationale for the difference in the dehydration conditions (e.g., within or outside the mold) is discussed next. Plugs were removed from each layer to measure the density, using ASTM D792-20, and the final thickness.

**Figure 1:**
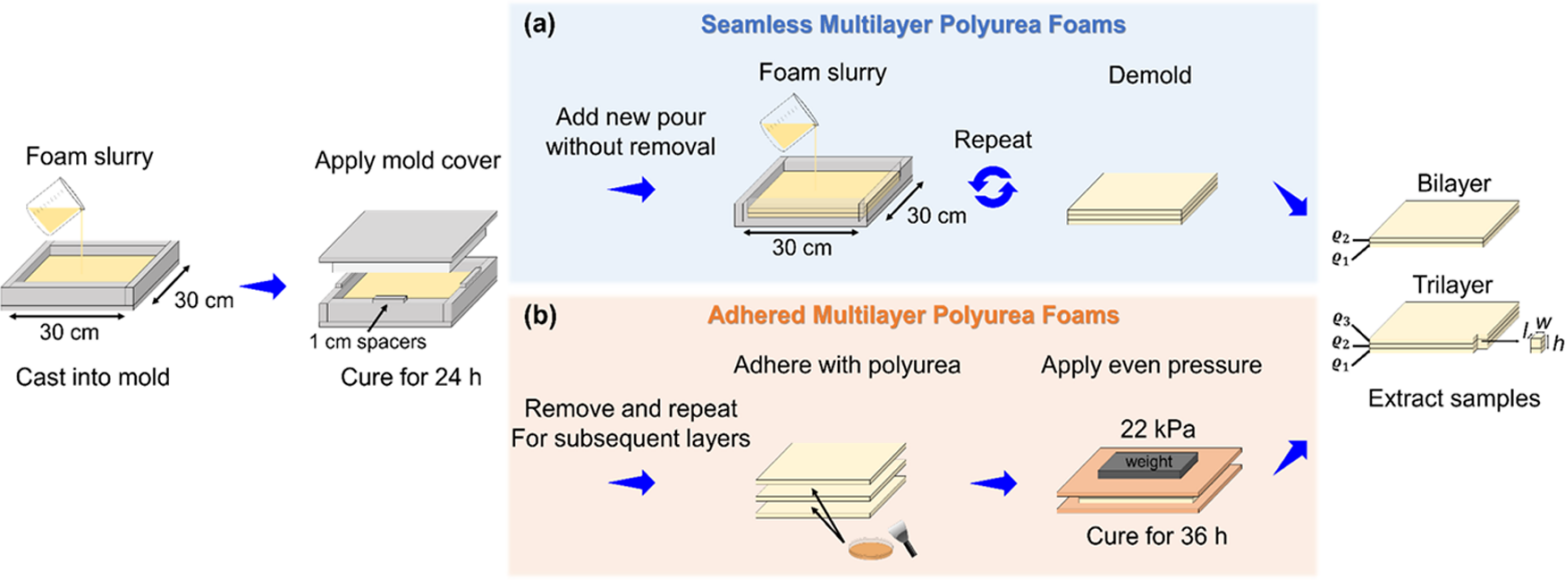
Summary of the sample preparation procedure, including (a) seamless interface and (b) adhered interface, density-graded polyurea foam sheets.

The first sample configuration is density-graded polyurea foam consisting of two or three layers without any foreign adhesive layer, relying on the natural adhesive property of the foam slurry, as discussed above. In the absence of an adhesive layer, these density gradients are referred to as a “seamless interface” given the intrinsic bonding between adjacent layers since they are made of the same base material. The foam manufacturing process discussed above was repeated twice to create bilayer, density-graded (hereafter referred to as “bilayer” for simplicity) foam sheets. The first layer was cast, cured, and dehydrated in the mold to avoid leaving any foreign contaminations on the surface that might hinder proper adhesion. Once ready, the second batch of foam slurry was poured onto the exposed surface of the cured foam. The same procedure was followed for creating trilayer, density-graded, polyurea foam sheets (hereafter referred to as “trilayer” for simplicity) by repeating the manufacturing process thrice. The thickness and density of the subsequent layers were controlled by adjusting the height of the cover/mold spacers and the pour weight. It is worth noting that a ∼25 mm strip was removed from the side of each layer before subsequent pours to facilitate the density measurements. At the end of the process, the density-graded sheets were removed from the mold and dehydrated for an additional 24 h before extracting five foam plugs from each fabricated sheet using a bandsaw. Figure 1a summarizes the manufacturing process to produce bilayer- and trilayer-graded sheets, including the dimensions of the foam plugs used in quasi-static compressive testing.

The second sample configuration also consisted of two or three layers assembled after complete curing and dehydration of the individual sheets. Once ready, the bonding surfaces were gently wiped with isopropyl alcohol to remove any residue that may have settled on the foam during idle periods. Next, a thin layer of bulk polyurea was applied to one of the surfaces (usually the lower-density foam) before assembling the graded sheets. Finally, the assembled structure was then cured under a constant pressure of 22 kPa between two rigid plates for 36 h. The polyurea adhesive was prepared by slowly and thoroughly mixing Versalink P1000 with Isonate 143L MDI in a 4:1 ratio for approximately 1 min. Since polyurea adhesive was used to assemble this configuration, it is denoted as “adhered” hereafter. Figure 1b recaps the manufacturing process of creating adhered bilayer- and trilayer-graded sheets.

**Figure 2:**
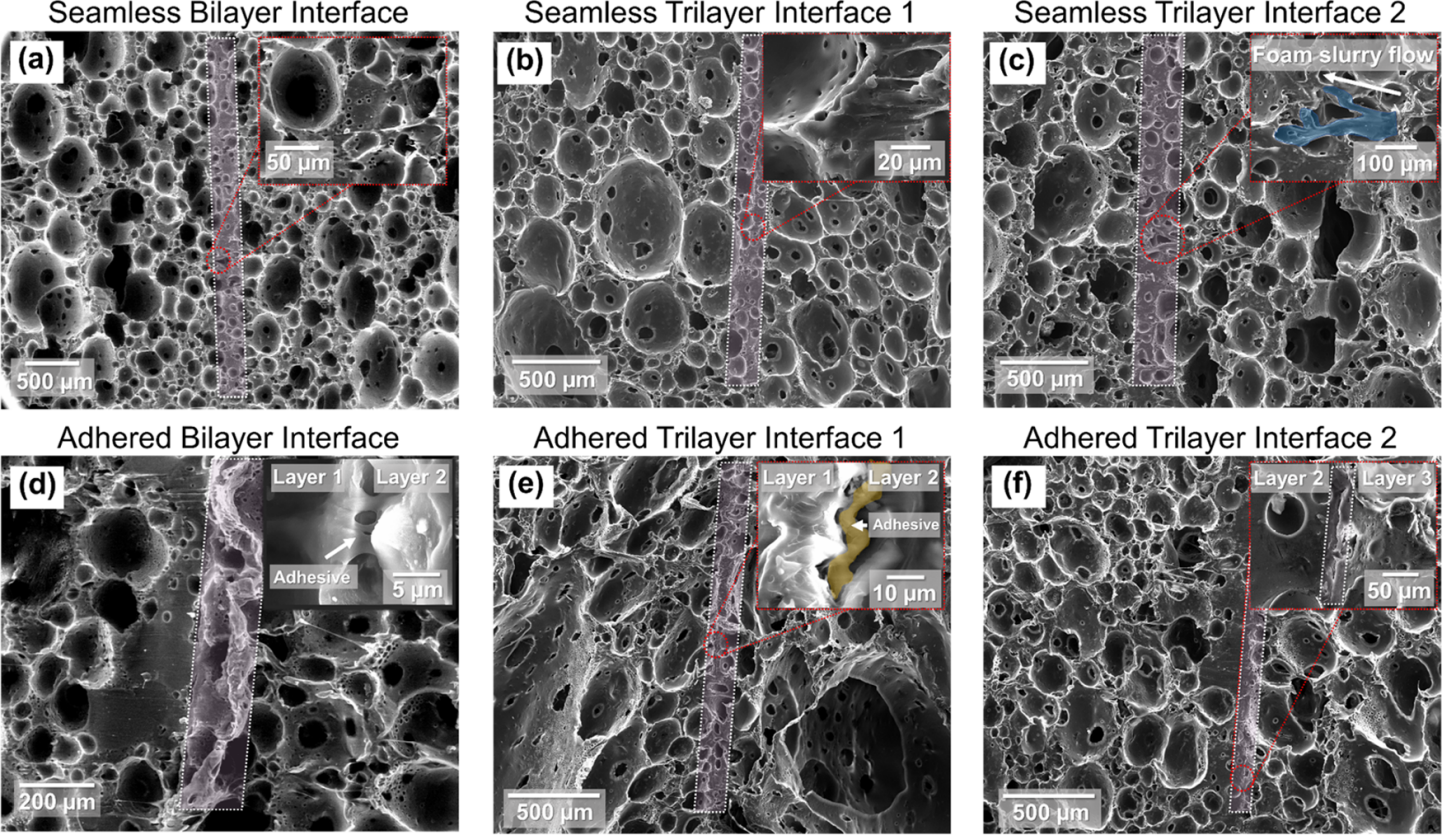
SEM micrographs highlighting the interfacial differences between seamless and adhered bilayer and trilayer density-graded polyurea sheets. For the bilayer interfaces, (a) shows a nearly indistinguishable interface while (d) exhibits a visible interface. For the trilayer interfaces, (b) and (e) correspond to the high- and medium-density foam ϱ_1_/ϱ_2_, while (c) and (f) correspond to the medium and low-density foam ϱ_2_/ϱ_3_.

Figure 2 is a collage of scanning electron microscopy micrographs showing the morphological differences between the adhered and seamless interfaces in bilayered and trilayered, density-graded foams. Analysis of the SEM micrographs reveals three insights about the density-graded elastomeric polyurea foams investigated herein. First, the micrographs from the seamless configurations, regardless of the number of layers, provide unequivocal evidence for the flawless transition from one foam layer to the next, exemplifying self-bonding after the sequential pouring of a fresh layer of polyurea foam slurry. While polyurea foam curing is an exothermal process, the increase in temperature was previously recorded to be ∼17 °C;^26,31^ such a low heat release rate during the curing process preserves the integrity of the chemical bonds, allowing natural bonding. This is contrary to other polymer foam technologies, e.g., polyurethane, where the heat-assisted curing process significantly increases temperature, affecting post-curing bonding and limiting the use of adhesives. The second insight is pouring the viscous foam slurry onto a cured polyurea foam sheet, resulting in the slow seeping of the soft, wet slime into the pores of the higher-density foam sheet. Notably, higher-density foam is characterized by smaller cell size,^24^ preventing full densification of the top layer by limiting the penetration process of the wet slurry. The penetration process is also hindered due to the quick set time for polyurea foams.^26,31^ The interpenetration of the slurry into the already cured foam provides additional improvement to the interface through mechanical interlocking. These two observations signify the success of fabricating multilayer density-graded elastomeric foam structures without adhesives or post-processing steps. Finally, the micrographs of the adhered multilayer foam samples indicate the ultrathin thickness of the adhesive layer, ∼5 μm, demonstrating an alternative and viable approach to achieve discrete gradation while minimizing the adverse effect of interfacing, as discussed in the forthcoming sections.

In addition to the density-graded samples, three control groups were also fabricated and tested. First, foam plugs were extracted from a single-layer polyurea foam sheet with a density of 255 kg/m^3^; these samples were denoted as “mono-density.” The mono-density samples were imperative to elucidate the effect of density gradation on the overall performance of polyurea elastomeric foams. The second control group consisted of samples extracted from a seamless bilayer sheet with comparable density but different layer thicknesses. Finally, the mechanical behavior of polyurea foams (inclusive of all of the configurations discussed above) was compared to a benchmark foam (ϱ^*f*^ = 395 kg/m^3^) that is commonly used as high-impact-mitigating padding in protective gears. Table 1 includes a summary of all of the sample configurations investigated herein.

**Table 1:** List of All Sample Configurations Investigated Herein, Including the Abbreviated Code, Density of Each Layer (ϱ_1_^*f*^/ϱ_**2**_^***f***^/ϱ_**3**_^***f***^ in kg/m^3^), Effective Density (ϱ^*f*^ in kg/m^3^), Length (*L*), Width (*W*), Height of Each Layer (*H*_1_/*h*_2_/*h*_3_), and Overall Height (*H*_o_)^a^

sample configuration	abb.	ϱ_1_^*f*^/ϱ_2_^*f*^/ϱ_3_^*f*^	ϱ^*f*^	*l*	*w*	*h*_1_/*h*_2_/*h*_3_	*h*_o_
seamless mono-density	SM	294/292/-	294	18.23	18.32	13.39/4.30/-	17.69
mono-density	M	255/-/-	255	17.82	18.38	17.49/-/-	17.49
seamless bilayer	SB1	332/287/-	311	18.04	18.74	9.83/8.91/-	18.74
	SB2	332/268/-	301	18.48	18.81	9.58/8.83/-	18.41
adhered bilayer	AB	309/253/-	281	18.42	18.71	10.28/10.26	20.54
seamless trilayer	ST	273/248/234	252	18.00	18.00	7.05/8.82/7.83	23.70
adhered trilayer	AT	309/253/231	265	18.43	18.53	9.85/10.56/10.14	30.35
D3O (benchmark foam)	D3O	397/-/-	397	17.01	17.17	14.18/-/-	14.18

a All dimensions are in mm.

### 2.2 Experimental Method

All quasi-static compressive tests were done in the force-controlled mode on a universal load frame (Instron 5843) with ±1 kN load capacity. The entire loading/unloading cycle was recorded with a peak load of 500 N and a loading rate of 250 N/min. The force-controlled mode was used in this study to avoid the early departure of the compression platen during the unloading portion of the cycle. This facilitated capturing nearly the entire hyperelastic response of the foam and quantifying unrecovered strains at no load since the recovery of polyurea foams is prolonged.^33,54^ The test was repeated five times on five samples (see Table 1) that had never been loaded. Virgin samples were used to avoid convoluting the results with fatigue, damage, and time-dependent deformation processes.^60^ The average compressive engineering stress–strain curves of each configuration, including the control groups and multilayer adhered and seamless samples, were calculated from all corresponding engineering stress–strain responses. The latter was individually calculated based on the undeformed cross-sectional area and original height of each sample.

### 2.3 Analysis Approach

The analysis approach emphasizes the loading portion of the stress–strain curves, which was applied to the average responses to implicitly account for intermeasurement variations. The analysis approach is divided into two regiments, including^1^ a set of discrete mechanical performance metrics and^2^ curve fitting of the average stress–strain curves into the Avalle model.^58^

The performance metrics used in this study were the tangent modulus to represent the mechanical stiffness, the specific absorbed energy capturing the strain energy dissipation capacity, the densification strain discerning the outset of the plateau region, the energy absorption efficiency, and the ideality. The latter two metrics were used to assess the performance of the foam in impact mitigation applications. The tangent modulus, , was calculated using the central difference method with *d*σ and *d*ε denoting the incremental stress and strain, respectively. The strain energy density, i.e., absorbed energy (*W*), was calculated as the area under the loading portion of the average stress–strain curve,
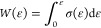
1

Since the homogenized stress–strain curves were reported for bilayer and trilayer configurations, the specific absorptivity (specific energy absorbed, SEA) was calculated by dividing the absorbed energy (eq 1) by the average density of their respective sample configuration.
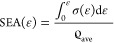
2

The densification strain was first estimated as the *x*-intercept of the line defining the tangent line at the maximum reported stress. It was also later calculated as the peak of the efficiency, as discussed next, indicating the onset of the densification region. Finally, to forecast the impact mitigation efficacy of the tested foam configurations, the energy absorption efficiency, η(ε), and ideality, *I*(ε), were calculated using eqs 3 and 4, respectively. The efficiency compares the performance of the foam to that of an ideal energy absorber counterpart, while the ideality is a measure of the ratio of absorbed and applied energies.^61,62^ Therefore, the strain at maximum ideality is associated with the optimal absorption performance, whereas the strain at maximum efficiency indicates the deformation condition for the largest energy absorption.
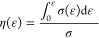
3
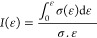
4

The individual regions within a typical stress–strain response of a foam sample, namely, elastic, plateau, and densification regions, can be readily captured by scaling laws as a function of the relative foam density.^63^ However, the homogenized stress–strain curve can also be represented by several empirical or phenomenological models, e.g., refs (59, 64, 65). The Avalle model was used herein to represent the average stress–strain curves of all investigated sample configurations
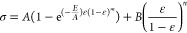
5where the first term signifies the elastic and plateau regions, while the second term embodies the densification.^59^ The material fitting parameters *A*, *E*, and *B* are density-dependent, while the exponents (*m* and *n*) are density-independent.^59^ The regression analysis was done in the Curve Fitting Toolbox in MATLAB using least squares regression algorithm while setting numerical bounds for each of the fitting parameters (eq 5) to accelerate the convergence process. The bounds included *A* ⊂ [0, σ̅], *E* ⊂ [0, 1] MPa, *B* ⊂ [0, ε_max_], *m* ⊂ [0, 1], and *n* ⊂ [1, ∞].

## 3 Results and Discussion

This section is divided into three subsections, delineating the results of quasi-static testing and the prediction of foam performance based on the mechanical response at a low strain rate. The last subsection is dedicated to the empirical model fitting and revealing insights about the behavior of the multilayer foam structures.

### 3.1 Quasi-Static Stress–Strain Responses

**Figure 3:**
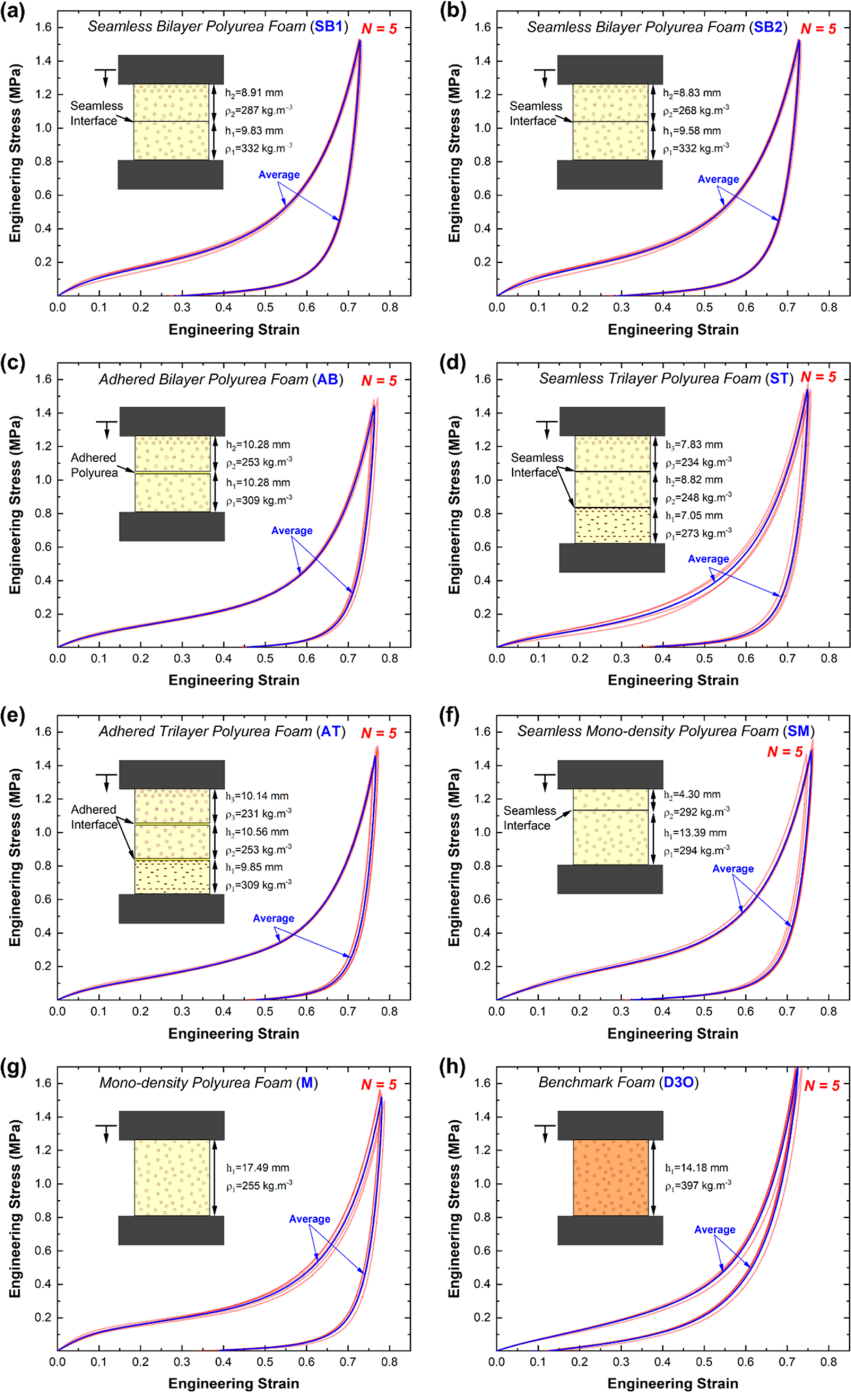
Stress–strain data of the quasi-static loading and unloading of each sample and their average for (a, b) seamless bilayer, (c) adhered bilayer, (d) seamless trilayer, (e) adhered trilayer, (f) seamless mono-density, (g) mono-density, and (h) benchmark foams.

**Figure 4:**
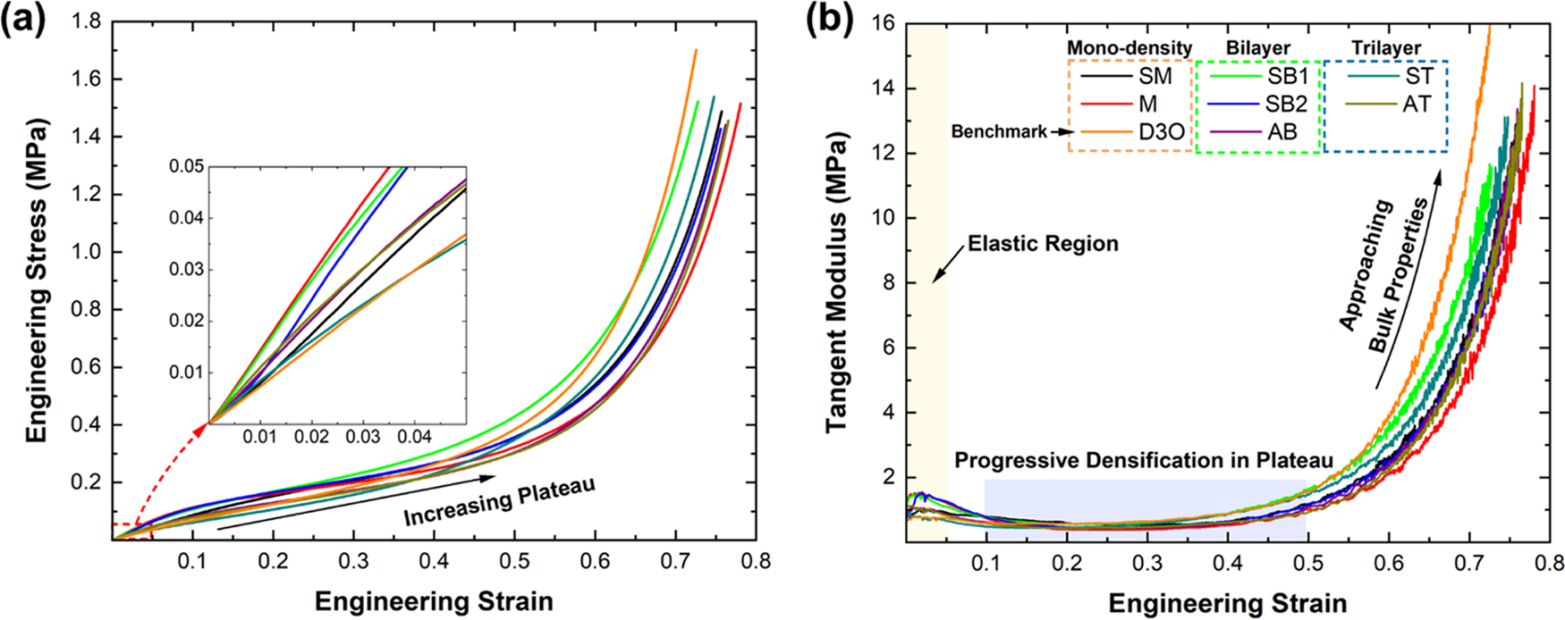
(a) Average stress–strain curves comparing the quasi-static mechanical behavior of seamless and adhered multilayer polyurea foams with control configurations and benchmark foams and (b) the corresponding tangent moduli.

Figure 3 contains composite plots of the engineering stress–strain responses of all sample configurations, comparing the mechanical response of seamless and adhered bilayer- and trilayer-graded polyurea elastomeric foams (Figure 3a–e) with that of control configurations (Figure 3f,h). The latter includes seamless bilayer samples (Figure 3f), where the density of both layers was nearly the same such that Δϱ*is merely 2 kg/m^3^, monolayer polyurea foam samples (Figure 3g), and the response of benchmark foam samples (Figure 3h). For each configuration in Figure 3, the average stress–strain behavior is plotted with all individual responses from the five tested samples for each configuration. The superimposition of the average and individual stress–strain curves signifies two outcomes. First, the overlap of the individual curves indicates high repeatability of the measurements, in turn, local conformity in the density throughout each of the fabricated polyurea foam sheets. In other words, the mechanical behavior of samples extracted from the same sheet closely resembles their adjacent counterparts, implying high intersheet consistency. Second, the high repeatability shown in Figure 3 led to low variations in each test. Therefore, the average of the five samples for each configuration represents the overall mechanical response. Hence, the average stress–strain curves are used hereafter to explicate the effect of interface type and the number of layers on the mechanical behavior of graded elastomeric foams. The average stress–strain curves (during loading half-cycles) are compiled separately in Figure 4a.

Three overarching and mutual observations are worth noting based on the results in Figure 3. First, the maximum stress is nearly the same, irrespective of the sample configuration, which is attributed to (i) the unified force history applied to all of the samples, as discussed in § 2.2, and (ii) the similar sample dimensions tabulated in Table 1. While the maximum force was preset at 500 N, all foam samples reported the three common regions consistent with the mechanical behavior of cellular solids, namely, the linear elastic, plateau, and densification. This justifies the initial selection of the amplitude of the applied force-time history. Also common to all of the mechanical responses in Figure 3 are the limited linear elasticity, extended rising plateau, and pronounced strain-locking densification regions. Second, the elastomeric behavior of the samples is exemplified in the pronounced hysteretic mechanical response, irrespective of the gradation or number of layers. The loading-unloading response demonstrates the reversible elasticity of these types of foam (some are delayed, as discussed next), while elucidating an intriguing twofold response. The benchmark foam exhibits a narrow hysteresis relative to all polyurea-based foam configurations, where the loading and unloading responses of D3O foam (Figure 3h) are nearly parallel and close. On the other hand, polyurea-based samples display a rapid spring-back upon unloading that is only restrained by the retraction rate of the compression plate during this phase, implying remnant strain energy within the foam continuum. That is, the mechanical response of the investigated foams follows, generally, a typical hyperelastic behavior. Finally, and a result of the second observation, is the temporary irrecoverable strain at the end of the loading cycle, exemplifying the time-dependent behavior of the foams investigated herein. All polyurea-based foams, irrespective of gradation or number of layers, reported temporary irrecoverable strain in excess of 30% as the compression platen departed from the sample surfaces, while the benchmark retained 10% at the end of the measurement cycle. It is important to note that all foam samples returned to their original height within a few minutes (<10 min) after load removal, denoting the irrecoverable strain as temporary.

Figure 4a captures the average stress–strain behavior of the investigated configurations, while Figure 4b reports the corresponding tangent moduli as a function of engineering strain. The results in Figure 4a reveal the effect of the gradation and interface type on the mechanical response of elastomeric foams. First, the responses of mono-density single (M) and bilayer (SM) samples with no density disparity are compared, showing that layering plays a role in the mechanical response. The average strain-strain response of the mono-density monolayer samples reported higher performance in the elastic region and the early plateau behavior (up to ε ≈ 0.25) compared to the bilayer counterpart. The former densified slightly after the mono-density, bilayer samples. Here, the difference in the foam density also mildly contributed to the difference in the mechanical responses of M and SM samples since Δϱ* is ca. 13%. Thus, layering resulted in a compliant response, affecting the efficacy of the foam, as discussed in the next section. A similar overall trend transpired when comparing the average response of the two sets of seamless bilayer samples (SB1 and SB2). The initial elastic and plateau regions of the seamless bilayer overlapped, showing no difference despite the persistence of a slight difference in average densities, i.e., Δϱ* < 4%. However, the response of these samples started to diverge in the latter part of the plateau region and into the densification region. The mechanical response of SB1 approached strain-locking before SB2, which reported ∼5% strain difference at 1.4 MPa. The change in the response, despite the close resemblance of the geometrical and gravimetric attributes of the samples, points to differences in the seamless interface quality that might have led to increased shear stress and adversely affected the strain transduction at the interface. This is the topic of companion investigations using full-field measurement to elucidate the deformation separation in these positively graded foam samples, even with such low gradation.

The interface type, being seamless or adhered, contributes to the mechanical behavior of bilayer and trilayer positively graded elastomeric foams. In the case of bilayer-graded samples, the average stress–strain response of the adhered samples was below that of seamless interface counterparts, reporting a mean difference of 2.2%. Notably, the adhered samples transitioned into the densification region at higher strain, where the corresponding densification strains for SB1, SB2, and AB are ε ≈ 0.60, ε ≈ 0.63, and ε ≈ 0.64, respectively. The densification strain was calculated using the method outlined by Ashby and Gibson^63^ by reporting the *x*-intercept of the tangent line to the flow stress. The delayed densification was also reported in adhered trilayer samples compared to their seamless companions, where the mean difference in the average stress–strain responses of seamless vs. adhered was merely 2.3% for ε < 0.4, increasing to 5.5% thereafter. The increased difference favors the adhered samples, broadening their mechanical performance, which is attributed to the presence of the ultrathin polyurea adhesive layer that instigates delayed strain transduction to the higher-density layer during compression and results in offset strain-locking densification. The lack of pronounced difference based on the interface is linked to (1) the thickness of the adhesive layer in the adhered samples (see SEM micrographs in Figure 2), (2) the adhesive and adherents being basically the same material, and (3) the relatively small difference in the gravimetric attributes in the gradation. Needless to say, at this point, the density (and variation thereof) played a role in the reported strain-strain behavior; however, the density contribution is convoluted given the sample structures and interface types.

It is now imperative to compare the mechanical performance of the graded samples, focusing on the seamless interface configuration, with the results of compressive testing of the benchmark foam. The results in Figure 4a suggest that gradation benefited the overall mechanical behavior of polyurea elastomeric foams with respect to the stress–strain behavior of the benchmark foam. Figure 4a (inset), which emphasizes the tangent modulus in the low-strain regime, indicates that the stiffness of the benchmark foam in the elastic region is inferior to all polyurea-based foam, irrespective of the gradation or interface type. Additionally, the mono-density and seamless bilayer foams reported higher strengths in the plateau region than the benchmark foam, implying enhanced stiffness and plateau performance. The bilayer foams, irrespective of their density, also outperformed the benchmark foam in the densification region and reported a delayed strain-locking behavior at slightly higher strain, indicating higher efficacy, as discussed in the next section. The trilevel gradation generally yielded a similar mechanical performance to the benchmark foam in all of the regions except a slight prominence in the densification region, where the former exhibited delayed densification compared to the latter. The delayed densification is attributed to the difference in the average density of the trilayer structures, seamless or adhered, with respect to the relatively high density of the benchmark foam. The mechanical resemblance between the performance of the trilayer-graded structures and the benchmark foam exemplifies the potential of graded polyurea foams to outperform the gold standard of impact-mitigating foams at lower weight penalty through strategic and optimal gradient designs. The latter is a topic of future research.

Finally, Figure 4b shows the tangent moduli vs. strain for all tested sample configurations, including the benchmark foam. Given the commercial characteristic of the benchmark foam (marketed as the gold standard in impact mitigation in motorsports applications), its plateau stiffness generally exceeded that of polyurea-based samples. In the densification region, the tangent moduli of polyurea-based configurations were comparable, while being consistently lower than the benchmark foam at any given strain in the region. It is worth noting that the maximum tangent modulus for reported polyurea-based foams is 6.7 ± 1.0 MPa (calculated at ε = 0.7), which is nearly 10% of the bulk polyurea modulus.^30^ The relatively low modulus in densification infers that higher stresses can be achieved before complete strain locking and flow, i.e., polyurea foams can provide protection for more severe loading conditions than those investigated herein. This research group is concurrently investigating the impact behavior of the same sample configurations discussed in this report, a matter for future disclosure. At the outset, the tangent modulus as a function of strain, Figure 4b, is offset from coincidence with the abscissa in the plateau region, departing from the idealized behavior of cellular solids where the stress remains nearly constant at an increasing strain till the onset of densification. On average, the tangent modulus was 0.55 MPa within the plateau region, extending from ca. 0.1 < ε < 0.4, which indicates that the plateau stress is monotonically increasing as a function of strain because of the progressive densification of all sample configurations studied herein.

### 3.2 Prediction of Impact Efficacy

**Figure 5:**
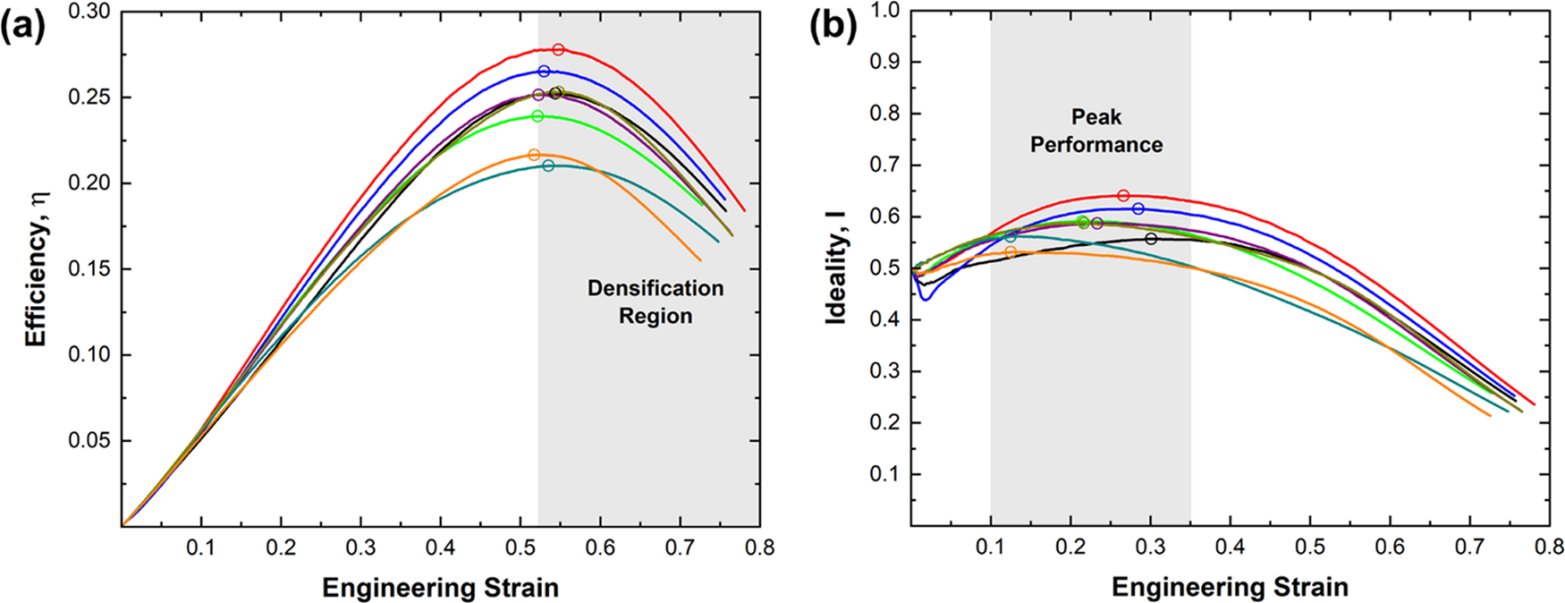
(a) Efficiency and (b) ideality for all investigated foam configurations with maximum efficiency and ideality marked by hollow circles, on their respective plots.

The efficacy of foams in impact mitigation applications can be forecast using several key performance indicators (KPIs), including the energy absorbed (*W* from eq 1), the specific energy absorbed (SAE, eq 2), the efficiency (η, eq 3), and ideality (*I*, eq 4). Table 2 summarizes these KPIs for all investigated sample configurations, showing that the mono-density, single-layer polyurea outperformed all other arrangements, including the benchmark foam, which is consistent with the outcomes of previous research.^26^ For the M sample configuration, the specific energy absorbed is 1095 J/kg, maximum efficiency is 0.28, and maximum ideality is 0.64 (Figure 5), leading over the benchmark foam with a significant margin despite being nearly 44% lighter. In other words, single-layer polyurea foam is a viable engineering contender to the industry standard incumbent, offering comparable load-bearing capacity and higher energy absorption, both at a lower density. The seamless trilayer (ST) polyurea foam sample configuration reported the highest SEA of 1014 J/kg, a 42% improvement over the benchmark foam. The compressive behavior of ST configuration prognosticates a maximum efficiency of 0.26 and onset of densification at ε_d_ = 0.54, calculated based on the strain corresponding to peak efficiency. However, the maximum ideality for ST configuration occurred at ε = 0.125, matching that of the benchmark foam. All other sample configurations reported maximum ideality at higher strains ε > 0.22. The delayed ideality implies a potentially higher performance of polyurea-based foam for severe impact scenarios that result in increased compression of the structure. This is further substantiated by the enhanced efficiency of polyurea-based foams and the corresponding onset of densification, as listed in Table 2. In closing, it is also well known that these key performance indicators overestimate the impact efficacy since they neglect to account for the inertial and strain rate effects, present in actual impact loading conditions. Hence, a companion investigation focuses on the dynamic behavior of the foams studied herein.

**Table 2:** Summary of Key Performance Indicators for Investigated Foam Configurations and Control Sets, Including the Absorptivity (*W* in J/Cm^3^), Specific Energy Absorption (SEA in J/kg), Peak Efficiency (η_max_), Ideality (*I*_max_) Densification Strain (ε_d_ from Figure 4a), and Onset Densification Strain (ε_do_ from Figure 5a)

sample configuration	*W*	SEA	η_max_	*I*_max_	ε_d_	ε_do_
SM	0.27	934	0.25	0.58	0.64	0.54
M	0.28	1095	0.28	0.64	0.68	0.55
SB1	0.29	912	0.24	0.59	0.60	0.52
SB2	0.27	908	0.27	0.62	0.63	0.53
AB	0.25	881	0.25	0.59	0.64	0.52
ST	0.26	1014	0.21	0.56	0.63	0.54
AT	0.25	935	0.25	0.59	0.66	0.55
D3O	0.26	665	0.22	0.53	0.62	0.52

A brief case study is presented here to demonstrate the utility of the results thus far in biomechanical applications, such as bone protection during lateral fall of the elderly, a common issue due to deteriorated mechanical and structural properties of bones.^66,67^ aging and senior population may suffer osteoporosis, affecting the density and mechanical properties of bones.^66,67^ During lateral falls, fracture of the greater trochanter is ubiquitous,^68^ which can be mitigated by cushioning the undergarments using foam paddings to protect the head of the femur from the incoming force upon impact.^69^ The foam padding performance can be explicated by constructing the cushion curves for the configurations discussed herein, using the method outlined by Mills in designing product packaging.^70^ In this case study, the average anthropometric data a U.S. adult woman is used to demonstrate the applicability of the cushion curve in designing hip protection pads, where the hypothetical subject is 1.63 m tall with 0.84 m hip height (vertical distance from the ground to the greater trochanter). The latter is used as the drop height in the proceeding calculations.

The cushion curve is used to assess the performance of polymer foams in protecting packages due to vertical drops during transport, a scenario like the biomechanical event of lateral falls considered herein. The cushion curve is constructed by dividing the maximum stress in a fall scenario by the static stress calculated from quasi-static testing. The static stress, stress at rest, is based on the progressive absorptivity at a maximum stress (σ_m_) such that
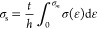
6where *t* is the foam padding thickness and h is the drop height.^70^ The ratio between the maximum and static stresses is the foam acceleration.^70^
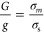
8

**Figure 6:**
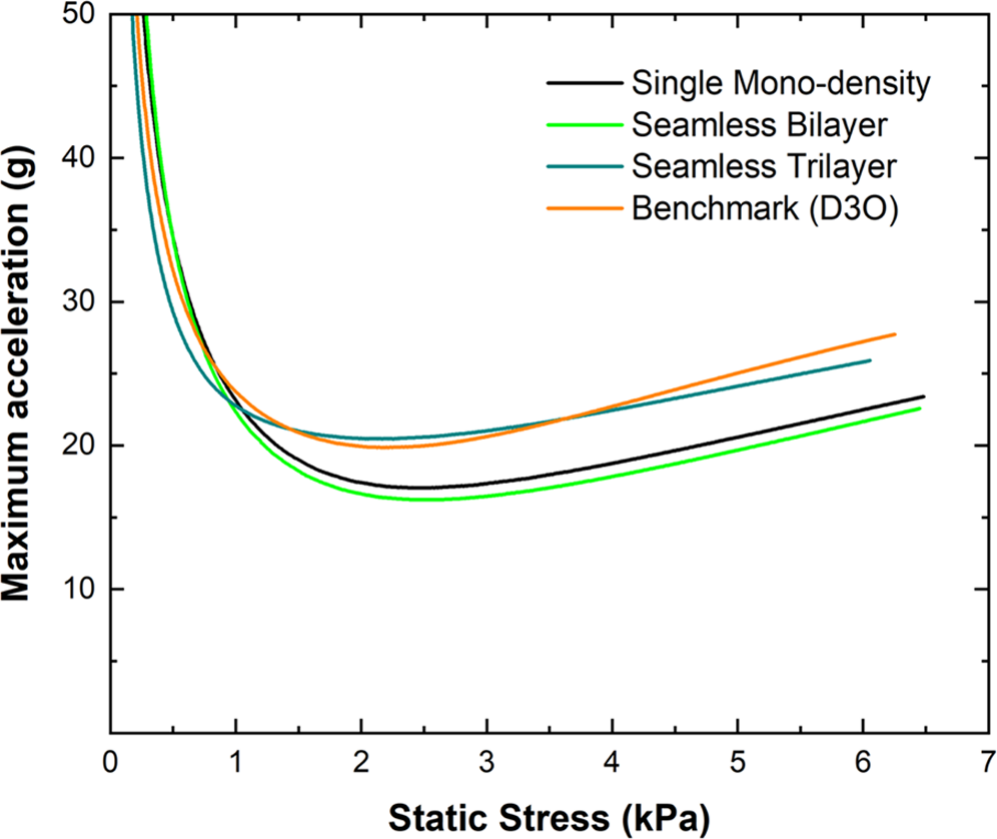
Cushion curves for all seamless polyurea foam configurations compared to the benchmark foams for 20 mm foam padding thickness sustain a lateral fall from 0.84 m height.

Figure 6 shows the predicted cushion curves for all seamless polyurea foam configurations, including seamless mono-density, bilayer, and trilayer, compared with the cushioning curve of the benchmark foam. Figure 6 was constructed by assuming a foam padding thickness of 20 mm and lateral fall from 840 mm height, as stated above. At low stresses, the cushioning performance of all foams is nearly identical, confirming the interchangeability of benchmark foam with a light polyurea alternative. At σ_s_ > 1 kPa, the seamless mono-density and bilayer polyurea foam samples outperformed all other configurations, reporting lower maximum acceleration and potentially offering superior protection against lateral fall without adding heavy and stiff foam padding to the sides of undergarments.

### 3.3 Semiempirical Model Results

**Figure 7:**
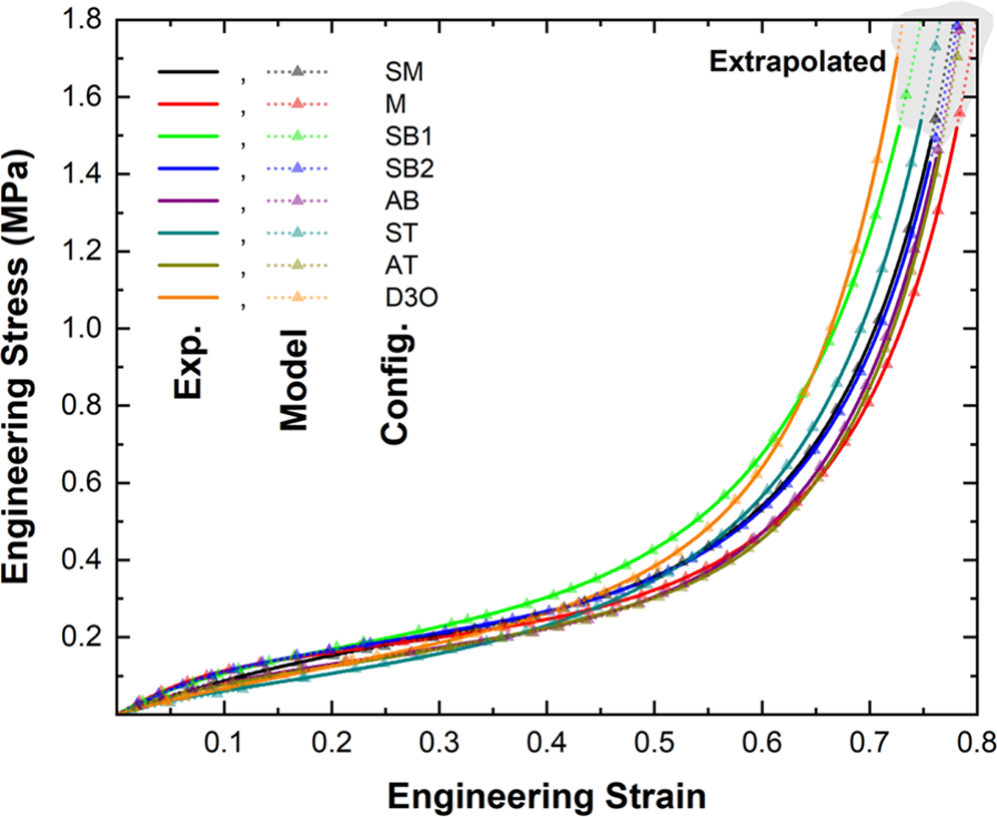
Avalle Model predictions using parameters from Table 3 for all averaged sample configurations and benchmark foam.

Figure 7 shows the comparison between the average experimental stress–strain curves and the Avalle-fitted counterparts with unity *R*^2^ values. Table 3 lists the resulting material fitting parameters, including the plateau stress (*A*), the modulus (*E*), and the densification modulus (*B*), shown to be density-dependent, as discussed in.^59^Table 3 also lists the strain exponents in the plateau and densification regions, *m* and *n*, respectively. The values of the elastic modulus are in reasonable agreement with the values of the tangent modulus in the elastic region shown in Figure 4b. The highest modulus was associated with the mono-density, single-layer polyurea foam at a value of 1.7 MPa, while the lowest modulus was associated with the seamless trilayer. Except for the former, the modulus of the benchmark foam was lower than all polyurea-based foam. The seamless bilayer foams reported high moduli compared to the adhered equivalent; however, reported low moduli when considering the trilayer seamless to the trilayer adhered. The modulus of the seamless bilayers is 28.3 and 33.4% higher than the adhered bilayer, while the modulus of the seamless trilayer is 44.4% lower than the adhered trilayer configuration. It is worth noting that the specific modulus for polyurea-based foams rivals its benchmark comparison since the latter has a higher density than all configurations of the former. In short, fitting the average stress–strain curves into the Avalle model provided some mechanistic insights into the mechanical behavior of the investigated foam configurations while revealing imperative mechanical properties, including *A*, *E*, and *B*.

**Table 3:** Fitting Parameters for Avalle Model Based on Average Stress–Strain Curves of All Considered Sample Configurations, with *A* (MPa) Corresponding to the Plateau Stress, *B* (MPa) the Densification Region Modulus, and *E* (MPa) the Elastic Modulus

sample configuration	*A*	*B*	*E*	*m*	*n*	*R*^*2*^
SM	0.21	0.17	1.14	0.00	1.89	1.00
M	0.18	0.14	1.69	0.22	1.76	1.00
SB1	0.22	0.23	1.46	0.57	1.77	1.00
SB2	0.19	0.17	1.54	0.00	1.78	1.00
AB	0.16	0.15	1.10	0.00	1.86	1.00
ST	0.24	0.20	0.63	0.48	1.78	1.00
AT	0.26	0.14	1.00	1.00	1.90	1.00
D3O	0.50	0.17	0.76	0.42	2.25	1.00

Conformal with the results shown in Figure 4a of the average stress–strain curves, the plateau stresses of nearly all polyurea-based foams fall lower than the benchmark counterpart, where the plateau stress (*A*) of the latter is nearly 9% higher than all polyurea-based foams irrespective of layering strategy or density. The increased value of *A* for the benchmark foam highlights the progressive densification behavior discussed in the previous section, where the average stress–strain curve of D3O ascends nearly monotonically till the onset of densification (e.g., ε_d_ = 0.52). The average plateau stress (*A*) for all polyurea-based samples was 200 kPa, indicating (1) an earlier transition into the plateau region from a distinct elastic regime and (2) a broad plateau region extending from 0.33 < ε < ε_d_. Similarly, the values of the densification modulus (*B*) conform with the results of the average stress–strain behaviors, where the lower values of *B* decreased the slope of the curve in the densification region, resulting in pushing the curves further to higher strains. The densification moduli of M, AB, and AT are 142, 148, and 140 kPa, respectively, also exemplified by extended stress–strain curves for these sample configurations. As the values of the densification modulus increases, the strain-locking shifted to slightly lower strains while taking into consideration the progressive densification behaviors and onset densification strains discussed above. Finally, the Avalle predictions signify the mild sensitivity to the plateau exponent, *m*, and strong sensitivity to the densification exponent, *n*, and listed in Table 3.

As mentioned above, the fitting parameters *A*, *B*, and *E* are known to be density-dependent, giving rise to correlation with their respective densities using Gibson scaling relations.^59^ Avalle et al. demonstrated the density interdependence of the plateau stress, elastic modulus, and densification modulus for single-density foams,^59^ showing excellent agreement between scaling law and their predictions. However, the average stress–strain curves reported in Figure 4a are convoluted by the individual responses of each layer within the graded structures, the density of each layer, and the interface type. Hence, we refrained from extrapolating the values of the Avalle fitting parameters based on Gibson scaling laws since the density value within each layer dynamically changes throughout the deformation history. Future research should emphasize amending the scaling law to account for the dynamic change in design at each strain level.

## 4 Conclusions

In closing, the research revealed two approaches to achieve discrete gradation of elastomeric polyurea foam, resulting in the fabrication of bilayer- and trilayer-graded structures for future impact mitigation applications. Polyurea foams had been previously investigated in nongraded or conventionally assembled techniques, leading to disparity in the response due to sizeable adhesive thickness. This study demonstrated that gradation with the seamless interface is technologically feasible and competitive with adhered bonding, even with ultrathin adhesive layers. Scanning electronic micrographs illustrated the quality of the seamless interfaces while evidencing chemical and mechanical bonding due to the sequential pouring of polyurea foam slurry to create the desired gradation. Foam plugs were extracted from several foam configurations, including monolayer and mono-density, seamless, bilayer mono-density, seamless bilayer- and trilayer-graded, and adhered bilayer and trilayer foam sheets, which were quasi-statically tested under compressive loading. The results were compared to the mechanical performance of closed-cell, mono-density benchmark foam considered the industry standard for impact mitigations. Polyurea foam, irrespective of gradation or interface type, outperformed the benchmark foam in several key performance metrics, potentially offering superior impact protection at a lower weight penalty. The results, in general, affirm the favorable influence of gradation on mechanical efficacy. The superiority of polyurea foams was explicated through a cushion curve to protect elders in lateral fall scenarios to guard against more significant trochanter fractures. The average stress–strain curves were also fitted into the empirical Avalle model with fitting correlation coefficients of perfect unity. The Avalle fitting parameters reveal essential insights into the plateau stress, elastic modulus, and densification modulus of all tested configurations. The outcomes of this research will guide future work toward affirming the superiority of graded elastomeric polyurea foams in absorbing impact energy in common biomechanical impact scenarios. The fabrication process unlocks the potential for continuously graded foam structures for impact mitigation applications.
